# 3D Printed Meter‐Scale Soft Origami Robots Capable of Load‐Bearing Actuation Via Crease Stiffness Modulation

**DOI:** 10.1002/advs.77127

**Published:** 2026-08-24

**Authors:** Sung Yol Yu, Sang‐Joon Ahn, Wonchul Lee, Seunguk Moon, Hyun‐Jin Hong, Kyu‐Jin Cho

**Affiliations:** ^1^ Soft Robotics Research Center Seoul National University Seoul Republic of Korea; ^2^ Department of Mechanical Engineering Institute of Advanced Machines and Design Seoul National University Seoul Republic of Korea; ^3^ Electronic and Hybrid Materials Research Center Korea Institute of Science and Technology Seoul Republic of Korea; ^4^ Shaping Matter Lab Faculty of Aerospace Engineering Delft University of Technology Delft The Netherlands

**Keywords:** 3D printing, fused deposition modeling, origami, soft robot

## Abstract

Origami has been a rich source for the design of soft deployable mechanisms capable of safe human‐robot interaction. However, the relatively weak structural stiffness of the folding crease lines gives rise to kinematic instability during shape transition, constraining practical robotic applications. Here, a 3D printed origami robotic actuator capable of load‐bearing deformation at the meter scale is proposed, enabled by programming the creaseline stiffness through modular assembly. During origami unfolding, the joint modules assembled onto the origami facets are stretched and subsequently provide antagonistic passive shape retention force to maintain kinematic stability. The integration of roll‐to‐roll (r2r) 3D printing further facilitates scalable fabrication of origami actuators with programmable robotic performance. The effectiveness of our framework is highlighted by a 1.3 m‐long collaborative robotic arm with variable workspace and an origami tent frame capable of automatic deployment and folding.

## Graphical abstract

Origami has inspired numerous soft deployable mechanisms for safe human‐robot interaction, while its intrinsic kinematic instability at arbitrary configurations limited their practical robotic application. This work presents a 3D printed meter‐scale origami robotic actuator capable of load‐bearing deformation through creaseline stiffness modulation. A collaborative robotic arm with variable workspace and a self‐deployable and foldable tent frame are demonstrated.

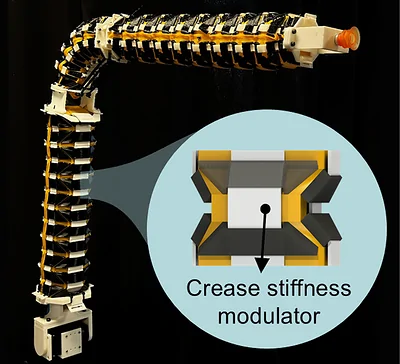

## 1 Introduction

To create robots capable of performing complex tasks alongside humans, space efficiency, safety, and payload capacity are the most critical considerations [1, 2]. Compared to traditional robotic arms composed of multiple rigid links and joints, soft deployable robots with versatile shape‐changing capabilities have recently emerged as a promising alternative [3, 4, 5, 6, 7]. The use of soft, flexible materials has imparted exceptional flexibility to soft robots, enabling the design of lightweight, space‐efficient machines with multimodal deformation capabilities [8, 9, 10, 11]. However, their inherent softness, while beneficial when safe interaction is paramount, significantly hindered their capabilities to withstand external forces, rendering them unsuitable for applications that require high load‐bearing capacity [12, 13, 14, 15].

Origami, an art of paper folding that transforms flat sheets into complex 3D geometries [15, 16, 17, 18], provides new opportunities for creating deployable robots. Origami structures can effectively support large external forces as the kinematic constraints between facets bear most of the stress at the deployed state, allowing high payload‐to‐weight applications. Moreover, various origami patterns, such as Miura tessellation [19], Yoshimura buckling [20], Tachi‐Miura tessellation [21, 22], and Kresling patterns [23, 24, 25] enable multiscale, multimodal deformations with high deployment ratio. Consequently, origami‐inspired designs have been extensively employed in various engineering domains such as inflatable architecture [26, 27, 28], self‐foldable machines [9, 25, 29], compliant robotic arms [7, 14, 22, 23, 30, 31], and variable‐wheel vehicles [32, 33]. The design and fabrication of foldable origami often rely on the laminated arrangement of flexible membranes with relatively stiff, constraining facets to achieve versatile shape‐shifting capability. As membrane origami is generally stable in the initial flat state, various techniques including mechanical deformation [16, 19, 25], thermoforming [22], and selective shrinking [34, 35] have been utilized to program a new equilibrium state in the folded or deployed configuration.

However, origami structures are intrinsically vulnerable to the nonuniform stress distribution at arbitrary configurations during shape transition. As a result, when external forces are applied at the arbitrary configuration, origami structures easily collapse or deform undesirably. To address this issue, various stiffness‐enhancing methods for origami‐inspired robots have been proposed for their real‐world deployment. Stimuli‐responsive materials such as shape memory alloys [25, 36] and shape memory polymers [37, 38, 39] show potential for selective stiffening, but they often suffer from slow actuation and environmental sensitivity. Thickness accommodation design methods [40, 41, 42] have been widely studied to achieve high structural stiffness at the meter scale. However, as a vast majority of these designs only exhibit load‐bearing capabilities in the deployed state, their practical application as robotic actuators is severely limited. Antagonistic stiffening methods that exert counterforces against origami folding are another approach for alleviating local stress concentrations along creaselines [22, 33, 43]. Nevertheless, these approaches not only necessitate complex multi‐actuator control, but are also limited in their ability to adaptively redirect force in response to dynamically changing origami folding angles. Soft origami actuators with rigid endoskeletons have demonstrated high payload capacity during pneumatic contraction; however, their actuation remains inherently unidirectional, precluding active deployment [44, 45, 46, 47]. Although pioneering studies have addressed such issues by integrating origami exoskeletons [14, 48] or utilizing multistability [26, 27], they are largely limited in their ability to systematically reconfigure origami kinematics and geometry for optimizing robotic performance. The emergence of 3D printing techniques has unlocked new possibilities for developing origami mechanisms by facilitating the programming of stress distribution with high precision, which is challenging to achieve with traditional methods [14, 18, 49, 50, 51, 52]. However, they often lack scalability due to their limited build volumes, while reprogramming the printed structures remains impractical. As robotic systems are often required to handle external loads during actuation, these limitations underscore the need for an origami programming strategy capable of preserving structural integrity and load‐bearing capacity across dynamically changing configurations.

Here, we present a 3D printed meter‐scale origami robotic actuator that exhibits high load‐bearing capability during shape transition, enabled by the assembly of antagonistic creaseline stiffening modules. We first show that the origami facets can be directly 3D printed onto a soft membrane, into which elastic, 3D printed joint modules are assembled to control local stiffness on the creaseline. When the origami actuator is pneumatically pressurized and deployed, the assembled joint modules stretch in tandem, providing an antagonistic shape retention force for each specific configuration without external control. We call this stiffening component creaseline stiffness modulators (CSMs). The strength of this approach lies in the fact that our origami actuator exhibits high load‐bearing capacity not only in the fully deployed state but also throughout its intermediate configurations (Table S1). This unique capability is exemplified by pneumatic deployment and retraction with ratios of up to 4.1 times and handling of weights of up to 10 kg while only weighing 400 g without the origami collapsing. Taking a step further, we integrate a roll‐to‐roll (r2r) feeding system into an FDM printer to enable direct 3D printing of origami patterns to create human‐scale soft origami robots, which is otherwise challenging to achieve with conventional origami programming methods. Taking advantage of this capability, we demonstrated a collaborative robotic arm with a variable workspace by integrating multiple actuators with tailored characteristics, enabling diverse tasks in close interaction with humans. By extending this modular concept, we demonstrated a self‐deployable and foldable origami tent frame that can be pitched up from 0.66 to 2.4 m in length, leading to a 35‐fold 3D volumetric expansion. Our framework may provide a scalable and effective pathway to develop soft origami robots that can be readily deployed to the real world.

## 2 Results and Discussion

### 2.1 Origami Folding Via Assembly of the Creaseline Stiffness Modulator

**FIGURE 1:**
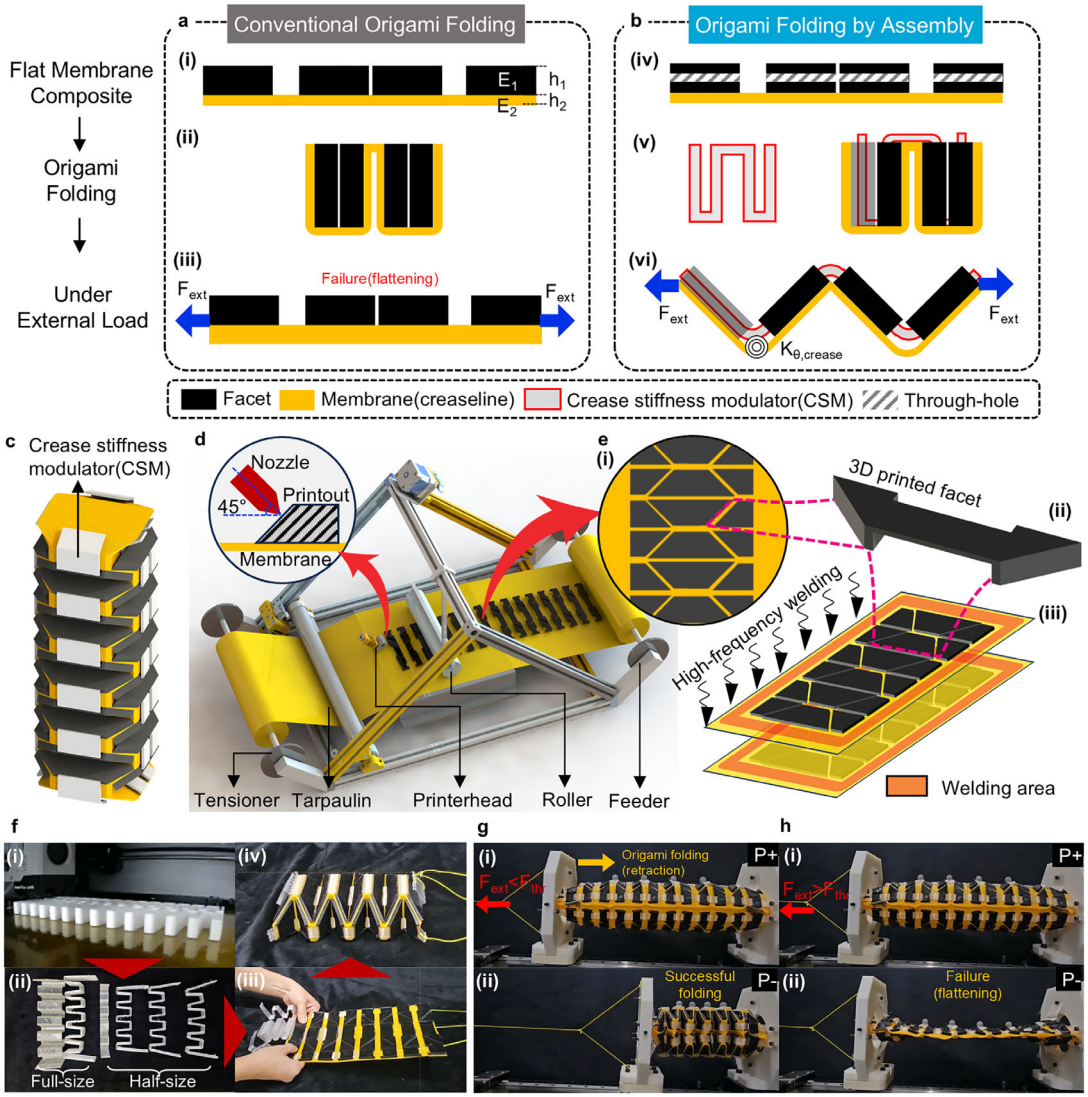
Origami folding by assembly of crease stiffness modulator. Schematics of (a) conventional origami folding (b) presented approach of folding by assembly. (c) Proposed origami actuator with assembled crease stiffness modulator (CSM). (d) Roll‐to‐roll (r2r) FDM 3D printing of origami facets on the flexible tarpaulin sheet. The motorized feeder, passive idler, pretensioning tensioners, and collection spool were integrated to ensure continuous and precise membrane feeding while maintaining a stable nozzle–membrane gap. (e) High‐frequency welding of 3D printed origami membrane to form an inflatable structure. (f) 3D printing and assembly process of CSMs. Load‐bearing actuation with payload (g) under the threshold leads to successful folding, whereas (h) payload above the threshold results in undesired flattening.

Traditional membrane origami fabrication processes often rely on the relative stiffness difference between the membranes and the facets to program the origami folding behavior (Figure 1). A creaseline embedded within a low‐stiffness membrane serves as a rotational joint connecting adjacent facets of relatively high stiffness (Figure 1a‐i, ii). For instance, mechanical folding weakens the membrane at the creaselines by deforming it beyond the elastic limit, creating localized plastic deformations [16, 19, 25]. Thermoplastic membranes can also be programmed into their folded states through thermal cycling and mechanical reshaping [22]. In 3D printed origami, the relative stiffness of the facets and creases is programmed through varying thicknesses or multimaterial printing with different elastic moduli [49, 50, 51, 52]. However, when exploiting relatively weak creaselines, the resulting origami structure becomes highly susceptible to external loads (*F_ext_*) in arbitrary configurations (Figure 1a‐iii). Otherwise, using membranes with high stiffness to support high payloads renders it almost impossible to program the desired folded state. In contrast, we assemble a passive, antagonistic spring on each rotational joint between the facets to achieve high stiffness along the creaseline while still allowing origami folding (Figure 1b). This crease stiffness modulator (CSM) is configured according to the fully folded geometry of the origami and is mounted along the through‐holes embedded in the facets (Figure 1b‐iv, v). Upon application of the external load, the CSM directly applies antagonistic torque to each crease arising from their elastic torsional stiffness, helping the origami preserve the deployment path defined by its kinematics. (Figure 1b‐vi). When the load is removed, the CSM provides restorative torque to facilitate desired folding behavior.

Using this origami design strategy, we developed a 3D printed pneumatic origami actuator that can be deployed and retracted while carrying external payloads (Figure 1c; Figure S1). Origami facets with through‐holes were directly 3D printed onto a soft, thin membrane to allow the assembly of CSMs for origami folding (Figure 1d; Movie S1). Unconventional origami design capability further emerges by integrating r2r feeding mechanism of a flexible membrane into the FDM printing system, enabling facile programming of origami patterns with a virtually infinite printable length (Figure S2). The target geometries were continuously printed with a relative angle of 45° between the printbed and the nozzle (Figure 1d, inset). Thermoplastic polyurethane (TPU) was used as the base printing material to allow stable origami deformation, whereas a TPU‐nylon‐TPU composite sheet (commonly referred to as tarpaulin) served as the membrane. A motorized feeding and tensioning system was implemented to ensure precise membrane transport while maintaining a consistent gap between the nozzle and the membrane. We employed Yoshimura buckling origami composed of equilateral trapezoids, in which four facets (N = 4) are connected at each vertex (Figure 1e‐i). To fabricate the structure from two membranes, each Yoshimura origami unit cell was designed with two full‐sized trapezoidal facets at the center and four bisected facets on both sides. Each 3D printed facet features an embedded tunnel, through which the CSMs are inserted and mechanically secured after they are inserted to the facet (Figure 1e‐ii). To print the overhanging tunnel ceilings of origami facets without using supportive structures, we optimized the printing parameters such as print speed, extrusion temperature, and cooling (see the Experimental Section). Otherwise, it would further complicate the fabrication process by adding post‐processing for removing supports. After being printed, an inflatable sealed structure was fabricated by stacking two membranes and melt‐bonding their TPU layers through high‐frequency welding (Figure 1e‐iii). The CSMs were also fabricated using a standard FDM printer and TPU base materials (Figure 1f‐i). Given the one‐to‐one correspondence of number of facets and CSMs, we employed two full‐size CSMs and four with half‐width dimensions (Figure 1f‐ii). Subsequently, the CSMs were manually stretched and inserted into the facet tunnels (Figure 1f‐iii). This process can be facilitated by attaching a thread to the opposite end and pulling it through in a weaving‐like manner. As a result, the origami actuator capable of pneumatically driven deployment and retraction under external payloads can be created (Figure 1f‐iv).

Upon depressurization for origami folding, the inflated membrane origami typically follows one of two pathways to reach the minimum volume (Figure 1g,h). When an external axial force (*F_ext_*) applied against the origami folding direction is smaller than the origami shape retention force (Figure 1g‐i), the origami maintains its prescribed folding path along the creaselines, leading to successful folding. (Figure 1g‐ii). In contrast, if the external force exceeds a certain threshold (*F_thr_*), the origami readily collapses along an undesirable energy pathway toward its initial flat membrane configuration, thereby hindering the linear contraction of the actuator. (Figure 1h). As the threshold shape retention force largely originates from the elastic torsional stiffness of the origami creaseline, local programming of this stiffness is essential for successful load‐carrying retraction of the origami actuator.

### 2.2 Soft‐Rigid Hybrid Kinematics and Payload Modeling of Yoshimura Origami

**FIGURE 2:**
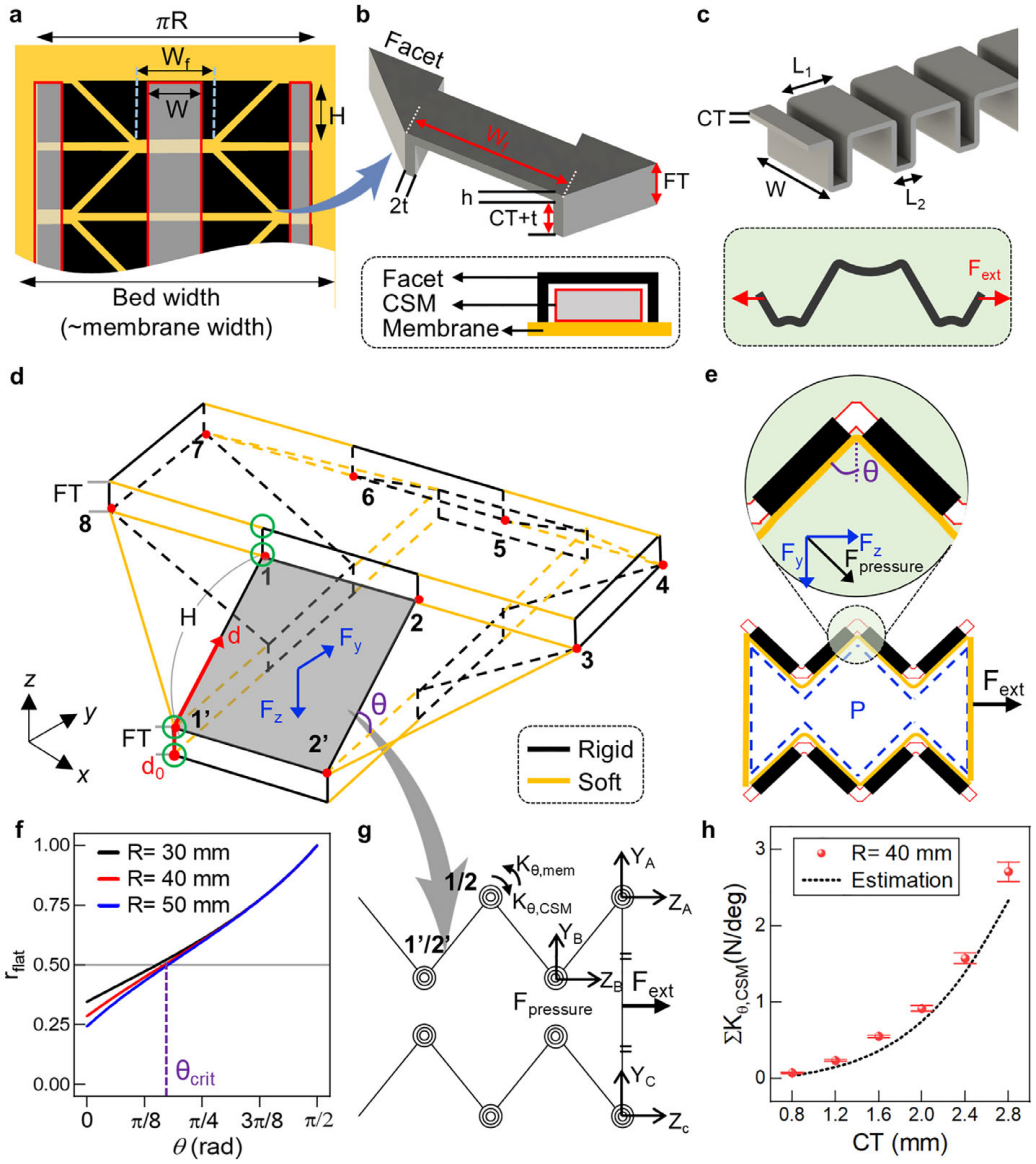
Yoshimura origami kinematics and payload equilibrium modeling. (a) Geometry of Yoshimura origami folding pattern (black shading) that is r2r FDM printed on the membrane (yellow shading). Gray shading indicates the through‐hole for CSM assembly. *R*, *W_f_‐*, and *W* denote the actuator radius, the shorter edge length of the full‐sized trapezoidal facet, and the full‐sized CSM width, respectively. (b) Geometry of 3D printed origami facet. *FT*, *CT*, *h*, and *t* denote the facet thickness, CSM thickness, thickness of the suspended ceiling in the facet, and clearance, respectively. (c) Design parameters of crease stiffness modulator (CSM). *L_1_* and *L_2_* denote the leg lengths corresponding to the mountain and valley crease, respectively. (d) Soft‐rigid hybrid kinematics model of buckling Yoshimura origami. A unit cell of N = 4 Yoshimura origami with trapezoidal facets is defined with soft (yellow) and rigid (black) edges. The green circles outline the vertices that are used for the kinematics. The vertices 1‐8 and 1′‐2′ are representative points that define the kinematics. (e) Simplified cross‐section of the origami actuator, illustrating the orientation of resultant force vectors from internal pressure. (f) The ratio of resultant force vectors oriented towards the folding and flattening directions as a function of the origami folding angle. Under negative pressure, the actuator begins to flatten if the origami folding angle exceeds the critical angle, *θ_crit_*, defined as the point where r_flat_ = 0.5 (i.e., when the two forces are equal). (g) Quasi‐static equilibrium model of the Yoshimura origami at ambient pressure. The rigid edges are modeled as links, while the CSM and membrane creases are represented as torsional springs with unit stiffnesses of *K_θ, CSM_* and *K_θ, Mem_*, respectively. (h) Total torsional stiffness of a set of CSMs corresponding to a Yoshimura unit cell with *R* = 40 mm as a function of creaseline thickness, *CT* (n = 5).

The direct origami 3D printing and reconfigurable assembly process offers a unique capability to precisely design origami actuators with tunable payload capacity. To systematically analyze this capability, we first introduced a soft‐rigid Yoshimura origami kinematic model (Figure 2). As Yoshimura origami originated from the buckling behavior of the cylinder [20], the origami actuator ideally deploys into a cylindrical cylinder with a radius of R (Figure 2a). Subsequently, the total width of the printed facets on a single membrane is set to be *πR*, while the trapezoid facet height, *H*, is determined by the trapezoidal angle (45° for the N = 4 Yoshimura pattern). To accommodate the facet thickness, the crease was enlarged by the *FT* in both, where *FT* denotes the facet thickness. Consequently, *H* was set to be πR/4‐2*FT*, while the shorter edge of the full‐sized trapezoidal facet was set to be *W_f_ = H+2FT*, and two bisected facets with the width of *W_f_/2* were placed on both sides. A suspended ceiling (i.e., an overhang) in the tunnel was designed with a fixed thickness of *h* = 1 mm across all specimens (Figure 2b). The overhanging gap between the ceiling and the membrane was adjusted based on the thickness of the CSMs with an additional clearance, *t*. To minimize friction during the CSM insertion into the tunnel, *t* was set to 1 mm. To ensure stable adhesion between the facets and the membrane, additional clearance of *2t* on each sidewall of the tunnel was added, leading to the tunnel width of *W_f_‐4t*. As a result, the tunnel height of 3.8 mm was used to allow the facile insertion of CSMs with a maximum thickness of 2.8 mm, leading to a total facet thickness, *FT*, of 4.8 mm. Given the facet geometries, the CSMs were designed with leg lengths *L_1_* and *L_2_* to ensure sufficient space to place facets between them during folding (Figure 2c). Specifically, the leg occupying a mountain crease has a length of *L_1_ = 2FT+t*, whereas the leg occupying a valley crease has length *L_2_ = L_1_‐2CT*. These legs contribute to bending stiffness as the CSM undergoes deformation under external loads (Figure 2c, inset). The width of the full‐sized CSM (*W*) was set to be *W* = *W_f_ ‐6t*, while the width of the half‐sized CSMs for the bisected facets was set to be *W_f_* /*2‐2t*.

A kinematic model of Yoshimura origami requires representation of the buckling behavior throughout deployment and folding [20]. As the soft deformation of the entire structure potentially introduces infinite degrees of freedom (DOFs), it is essential to localize deformations by categorizing the origami tessellations into soft and rigid edges [53]. The rigid edges define the global kinematics through a unique origami folding angle, *θ*, while the soft edges deform in response to the motion of the rigid edges. Starting from the fully folded state, the rigid edges deploy with the origami angle toward the final octagonal cylinder configuration, and the adjacent soft edges deform accordingly (Figure S3; Movie S3). At an arbitrary cross‐section of the Yoshimura unit cell, the rigid edges (1–2, 3–4, 5–6, 7–8) form the central quadrilateral of each facet translate purely without rotation during origami folding and unfolding. The length of the edge 1‐1′, which connects both ends of the origami unit cell is identical to the height of the trapezoidal facet, *H*. To accommodate the facet thickness, cross‐sectional kinematics were introduced over the intervals *0 ≤ d ≤ H+2FT* along the edge indicated by the red line in Figure 2d, where *d* denotes the offset from one end. The developed kinematics model can be used to calculate the locations of the eight vertices of any arbitrary cross‐section (located at an arbitrary *d*); however, the free‐body diagram and critical angle calculations only require 4 different cross‐sections (*d = 0, FT, H+FT, H+2FT*, indicated by green circles*)*. Consequently, the origami kinematics of each vertex can be mathematically represented by *H*, *d*, and *θ* (see Supporting Information).

When depressurized, the origami actuator experiences pressure loading normal to its internal facet surfaces, reaching its minimum volume state (Figure 2e). The undesirable origami collapsing towards its initial flat membrane state is triggered by a transverse component of the pressure loading, *F_y_*, which acts perpendicular to the origami folding direction. Conversely, successful origami folding can be achieved when a longitudinal component that drives folding, *F_z_*, exceeds *F_y_*. Therefore, the ratio of *F_z_* and *F_y_* at the onset of the negative pressure governs the behavior of the actuator. This ratio is purely defined by the ratio of the projected areas of the internal facet surfaces, *r_flat_*, which can be calculated by partitioning the internal surfaces of the origami into triangular surfaces and projecting them onto the corresponding directions (Figure S4). Therefore, the threshold origami angle, or the critical angle, *θ_crit_*, beyond which the flattening occurs immediately upon application of the negative pressure, can be analytically calculated by the actuator geometry that satisfies *r_flat_* = 0.5 (Figure 2f).

Building on this observation, we further developed a quasi‐static equilibrium model by constructing a free‐body‐diagram at the ambient pressure (Figure 2g). The rigid edges were modeled as links, while the stiffness of each origami crease was represented using a torsional spring. The torsional spring stiffnesses were derived by modeling the membrane and the CSM as bending beams with a uniform radius of curvature, expressed as follows:

(1) $$K_{\theta ,CSM}=\frac{E_{CSM}CT^{3}W}{12}\left(\frac{1}{L_{1}}+\frac{1}{L_{2}}\right)$$

(2) $$K_{\theta ,MEM}=\frac{E_{Mem}t_{Mem}^{3}}{12}\left(\frac{4\left(H+2FT\right)}{L_{1}}+\frac{\sqrt{2}\left(H\right)}{L_{2}}\right)$$

where *K_θ, CSM_*, *E_CSM_* denote the torsional stiffness and elastic modulus of the CSM, respectively, and those of the membrane, and *K_θ, Mem_*, and *E_Mem_* denote those of the membrane. Notably, the total torsional stiffness of both the CSMs and the membrane in a single origami unit cell can be obtained by multiplying the stiffness by 4, accounting for two full‐size CSMs and four with half‐width dimensions. To verify this, we prepared a set of CSMs with different *CT* values ranging from 0.8 to 2.8 mm, tailored to the origami actuator with *R* of 40 mm (Figure 2h). The thickness of the membrane was fixed at 0.43 mm across all samples. To measure *K_θ, CSM_*, a single unit of the CSM composed of one mountain crease and one valley crease (corresponding to *L_1_* and *L_2_*, respectively) was prepared and tested on a uniaxial tensile tester (Figure S5). The results show good agreement with the estimated values obtained from Equations (1) and (2). When assembled, the origami structure reaches a state of strain energy equilibrium between the folded and deployed extremes, represented by the intersection of the two energy landscapes (Figure S6). Consequently, the maximum axial payload, *F_ax, max_*, which corresponds to the threshold external force that pulls the actuator beyond *θ_crit_*, can be defined as the following (see Supporting Information):

(3) $$F_{ax,max}=\frac{2\left[\left(K_{\theta ,CSM}\right)\theta_{crit}+\left(K_{\theta ,MEM}\right)\left(\frac{\pi }{2}-\theta_{crit}\right)\right]}{\left(H+FT\right)cos\theta_{crit}}$$

### 2.3 Tunable Performance of 3D Printed Origami Actuators

**FIGURE 3:**
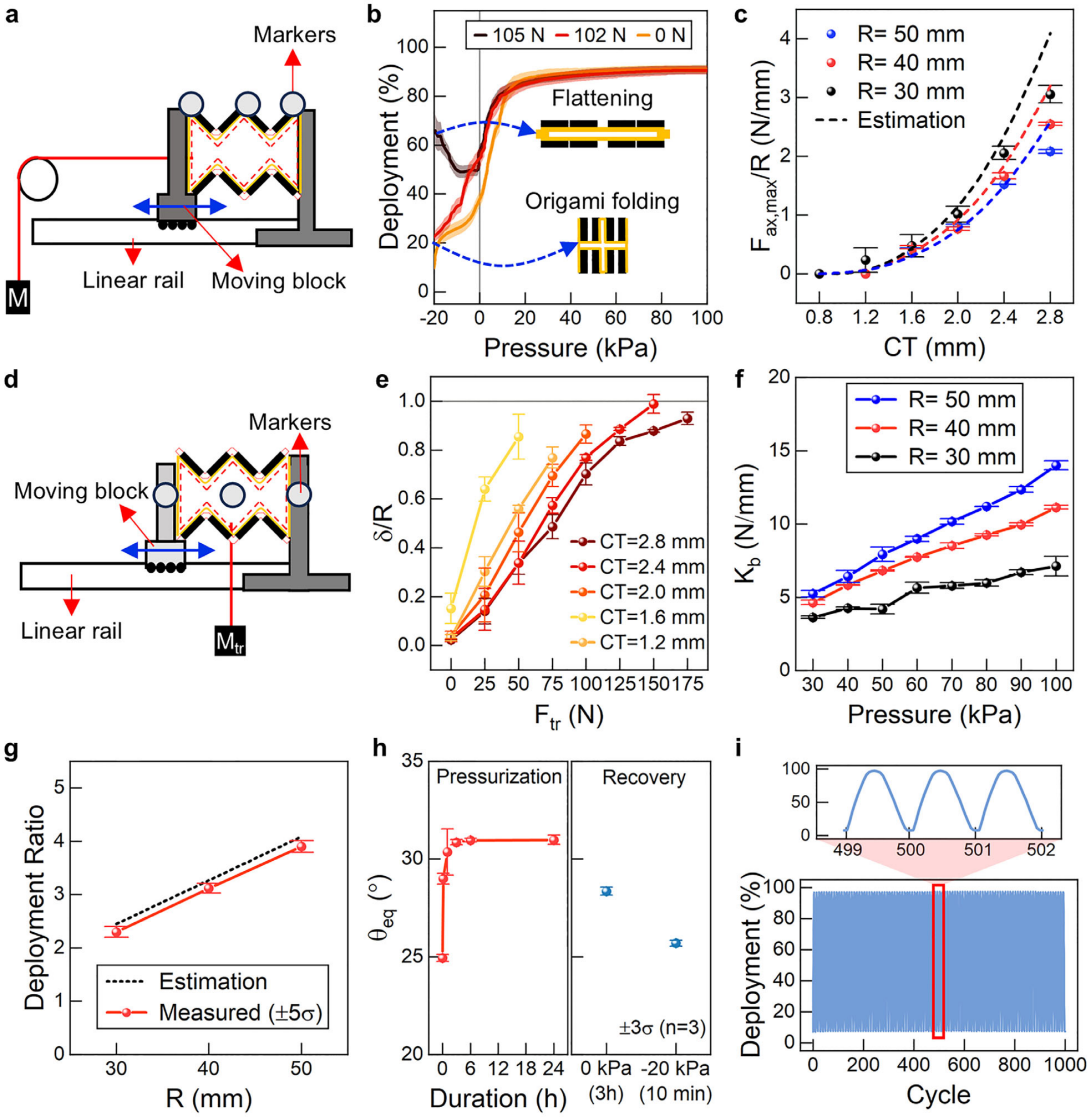
Performance characterization of the origami actuator. (a) Schematic of the axial payload test setup. Reflective markers were placed on each mountain fold to capture the origami folding trajectories. (b) Deployment of the origami actuator as a function of applied pressure (n = 3). Deployment was normalized between the designed fully deployed length of the origami unit cell (100%) and its folded length (0%). The actuator exhibits rapid volumetric shrinkage near ambient pressure, followed by either successful folding or collapse depending on the applied load. (c) Normalized maximum axial payload, *F_ax, max_* /*R*, as a function of creaseline thickness (n = 5). (d) Schematic of the transverse payload test setup. A weight was suspended at the center of the actuator, and the motion capture system measured the flexural deformation. (e) The normalized flexural deformation of the actuator, *δ/R*, as a function of the applied transverse load (n = 5). (f) Pressure‐dependent bending stiffness of the fully deployed origami actuator (n = 3). (g) Deployment ratio as a function of radius (n = 3). (h) Long‐term durability test result. The actuators were pressurized to 100 kPa for given durations, exhibiting gradual degradation in the equilibrium angle. Subsequent depressurization resulted in the full recovery of the initial *θ_eq_* value of the actuator. (i) Cyclic test for the origami actuator.

The origami actuators with CSMs exhibit a dynamic load‐bearing capacity during pneumatic actuation (Figure 3). The linear actuation of the load‐carrying origami actuator was measured using a motion tracking system (Figure 3a). Although the welded airtight enclosure remained intact over a pressure range of −80 to 200 kPa, the origami actuator was operated using a simple binary control scheme, with 100 kPa for deployment and −20 kPa for retraction, to ensure stable operation. The deployment was normalized between the designed fully deployed length of a Yoshimura unit cell (*H+2FT*) and its folded length (*2FT*). The origami actuator maintains its deployed configuration across a wide positive‐pressure range, exhibiting rapid folding only when the internal pressure approaches atmospheric pressure (Figure 3b). In contrat, under a substantial axial payload, the onset of negative pressure induced collapse toward the flat membrane state, resulting in an increase in deployment. To evaluate the load‐bearing capacity, we defined the maximum axial payload as the largest payload under which the actuator could successfully deploy and retract without collapsing. The origami actuators with different *R* values ranging from 30 to 50 mm and corresponding CSMs with different *CT* values ranging from 0.8 to 2.8 mm were prepared and tested (Figure 3c). The maximum axial payload, *F_ax, ma_*, was normalized with respect to *R* for comparison. Both the estimated values obtained from Equation (3) and the measurements showed consistent results, revealing an exponential increase in maximum payload with increasing *CT* values. Notably, the performance of the origami actuator with *R* = 50 mm and *CT* = 2.8 mm was maintained for axial loads up to 102 N, corresponding to *F_ax, max_*/*R* = 2.08. The origami actuator with *R* = 30 mm consistently demonstrated higher *F_ax, max_*/*R* values up to 3.06 than those with larger radii. These results suggest that workspace efficiency and fabrication cost of the actuator can be tailored by adjusting R.

The transverse load‐bearing capacity of the origami actuator during dynamic configuration changes was also evaluated (Figure 3d,e; Movie S4). The flexural deformation of the origami actuator under a transverse payload applied at its center was measured during the pneumatic actuation (Figure 3d). The performance of the origami actuator with *R* = 50 mm was evaluated under the transverse payloads in increments of 25 N while using CSMs with increasing *CT* values (Figure 3e). The maximum transverse payload (*F_tr_)* was defined as the largest payload under which the actuator could operate before either collapse occurred or the normalized flexural deformation, *δ/R*, reached 1. The origami actuator with *R* = 50 mm and *CT* = 2.8 mm achieved successful deployment and retraction under a transverse payload up to 175 N, while *δ/R* increased to 0.93. The bending stiffness (*K_b_*) of the origami actuator, which governs its static load‐bearing capacity in the fully deployed state, can be modulated through both actuator design and operating pressure (Figure 3f and Figure S7). We used a set of origami actuators with varying *R* values ranging from 30 to 50 mm, while maintaining a constant *CT* of 2.8 mm. The actuators were tested over a pressure range of 30–100 kPa, where their configurations had effectively converged to the fully deployed state. The three‐point bending test results show that *K_b_* gradually increases with pressure, reaching its maximum value of 14 N/mm at 100 kPa for R = 50 mm. In addition, comparison with actuators without CSMs suggests that CSM integration slightly increases the effective flexural rigidity of the actuator (Figure S7c). Furthermore, as shown in Figure 3g, the deployment ratio of the origami actuator can be tuned by changing the radius, as follows:

(4) $$r_{deploy}=\frac{\pi R}{8FT}$$

The maximum deployment ratio of 3.96 was obtained with *R* = 50 mm, consistent with the analytical estimations. Although the overall facet thickness was fixed at 4.8 mm regardless of the *CT* values in this study, it is noteworthy that thinner CSMs could allow further reduction in facet thickness and thereby increase the deployment ratio.

Taking this step further, we tested the long‐term durability of the proposed actuator as shown in Figure 3h. A set of origami actuators with R = 40 mm and CT = 2.8 mm were pressurized to maintain the fully deployed configuration. After durations of 10 min, 1 h, 3 h, 6 h, and 1 day, actuators were depressurized to the ambient pressure to measure the equilibrium angle. The result showed that the equilibrium angle gradually increased over time, suggesting a time‐dependent degradation of the antagonistic shape retention force of the CSMs. This degradation can be attributed in part to the viscoelastic relaxation of the CSMs, as the equilibrium angle was partially restored after resting the actuator at ambient pressure for 3 h. Further retraining at −20 kPa for 10 min fully recovered the initial equilibrium angle of 25°, suggesting that the accumulated residual deformation of the CSMs can be readily reprogrammed through reverse loading. Notably, the modular assembly concept allows facile replacement of defective CSMs on demand. Moreover, stable deployment and folding were maintained over 1000 cycles, demonstrating a robust performance of the origami actuator (Figure 3i). The origami actuator with a radius of 40 mm and length of 300 mm was pressurized up to 100 kPa and then depressurized down to −20 kPa with a sufficient interval to prevent overheating of the pumps. As a result, the actuator operated reliably without noticeable degradation in deployment performance.

### 2.4 Soft Origami Robotic Arm with Extensive Reachability

**FIGURE 4:**
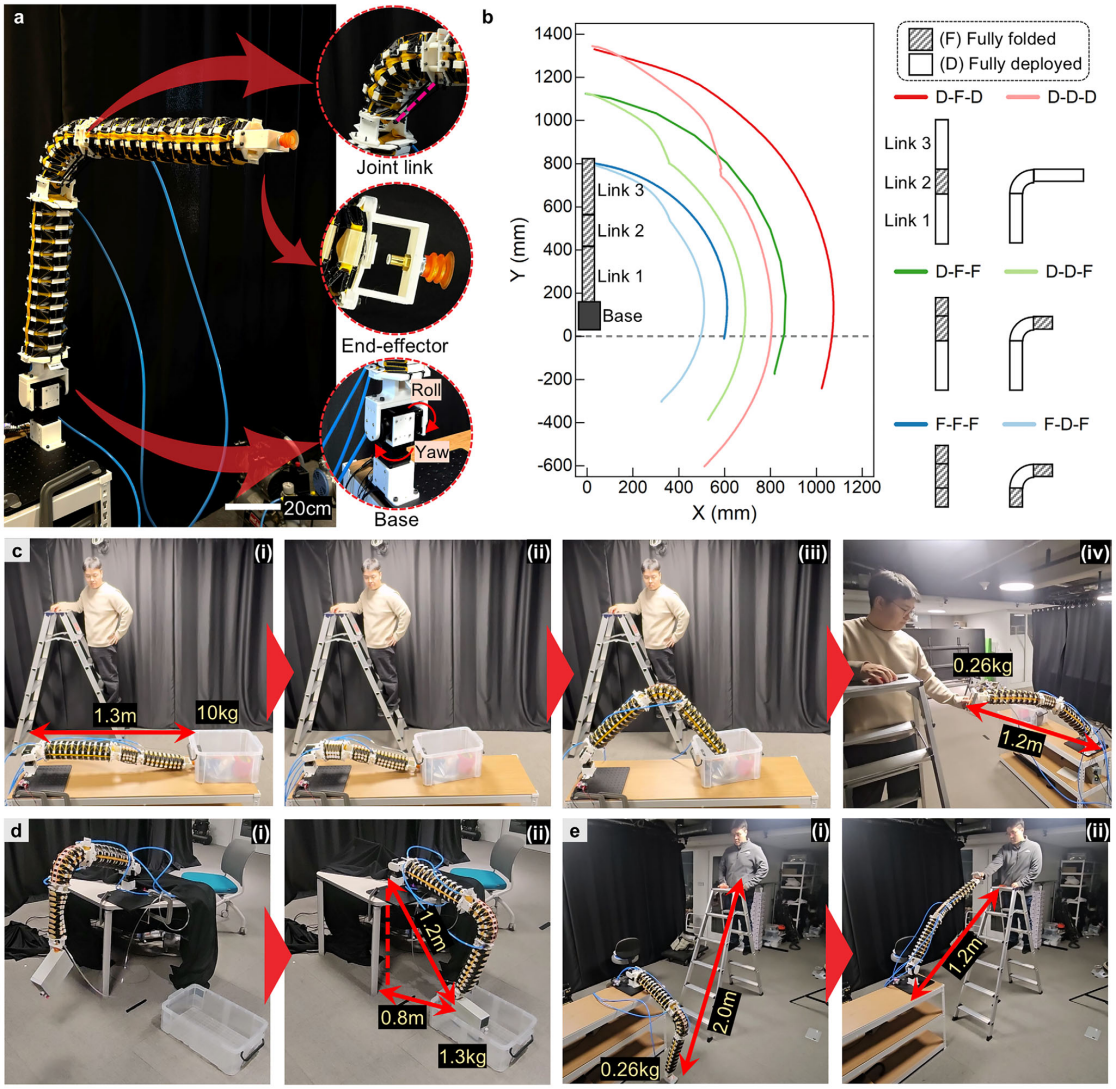
Soft origami robotic arm with variable workspace. (a) Origami robotic arm comprising two linearly deployable links and one bending link. The base electric motor provides yaw and pitch motions, while a commercial suction cup is mounted at the distal end. (b) The trajectories of the robot arm under different link configurations. Each origami link is independently operated using binary pneumatic control, where D and F denote fully deployed state at 100 kPa and fully folded state at −20 kPa, respectively. (c–e) Collaborative operation including load carrying, bin picking, and human robot interaction.

To highlight the capabilities of the 3D printed origami actuator with CSMs, we fabricated a multi‐DOF soft robotic arm capable of on‐demand deployment and retraction to modulate the workspace (Figure 4). Soft robots have drawn substantial attention as a promising alternative to the conventional rigid manipulators due to their ability to perform dexterous interaction and stretch over an extensive range [3, 4, 5, 6, 7, 8, 9, 10, 11]. However, the trade‐off between flexible deformability and load‐bearing capacity, which stems from the inherent softness, remains a significant challenge for practical applications.

Using our origami design and printing strategy, we developed the robotic arm composed of three deployable origami links (Figure 4a). The proximal and distal links that undergo linear actuation have radii of 50 and 40 mm, and deployable lengths of 460 and 530 mm, respectively. The intermediate link, which provides a bending motion, has a radius of 40 mm and length of 290 mm. To achieve the bending motion of the origami actuator, one side of the actuator was constrained using the cable tie (highlighted with a red dotted line) with limited length (Figure 4a, inset). Owing to the high load‐bearing capacity, the origami actuator can sustain operation without mechanical failure even when one end is completely constrained against deployment, resulting in bending toward the constrained side. Meanwhile, the rotational motions in the yaw and pitch directions were driven by electric motors, and a commercially available suction cup was mounted as an end‐effector (see the Experimental Section). Each origami link and the end effector were pneumatically connected in parallel to a single pump, allowing independent actuation (Figure S8). Notably, the body of the robotic arm is inherently soft, enabling safe interaction with the environment compared to its conventional, rigid counterparts.

To demonstrate the workspace modulation capability, the trajectories of the robotic arm were evaluated under different pneumatic actuation modes (Figure 4b, Movie S5). Each origami link was selectively pressurized to 100 kPa for deployment, or depressurized to −20 kPa for retraction, resulting in six distinguishable modes. From its fully retracted length of 810 mm, including the base motors and mount, the robotic arm can extend up to 1345 mm, exhibiting a 4.6‐fold increase in volume. Furthermore, it can reach 600 mm below its base, enabling object manipulation from the floor while being installed on a table. In addition, the robotic arm demonstrated high positional repeatability over 50 cycles, exhibiting RMS radial distances of 0.588 and 0.559 mm at the bottom and top endpoints, respectively (Figure S9).

This unique functionality enables a range of cooperative scenarios that are otherwise challenging to achieve with existing rigid‐bodied manipulators (Movie S6). As illustrated in Figure 4c, the robotic arm (i, ii) approached and pulled a 10 kg toolbox located 1.3 m from its base, (iii) retrieved a 0.26 kg tool from the box, and (iv) delivered it to an operator at a straight‐line distance of 1.2 m. Moreover, it can perform a pick‐and‐place task by (i) grasping a 1.3 kg object and (ii) moving it to another location positioned 1.2 m radially and 0.8 m vertically below the base (Figure 4d). Notably, the proposed robotic arm sustained a payload‐induced torque of approximately 10.2 N m, calculated with respect to the base, which slightly exceeds the rated torque of the base electric motor. In addition, as shown in Figure 4e, (i) a 0.26 kg tool placed at the same relative distance from the base was (ii) transferred to a user located 2 m away (i.e., 1.2 m from the base). These diverse human‐robot interactions are ensured by the intrinsic softness of the robotic arm, as evidenced by a contact pressure of 60.2 kPa at an end‐effector speed of 3 m/s, which remains far below the permissible transient contact pressure limits ranging from 110 to 600 kPa (Figure S9). Collectively, these results demonstrate the remarkable capability of the proposed robotic actuators to perform real‐world tasks, enabled by the high load‐bearing capacity and deployability achieved through CSM‐assisted origami folding.

### 2.5 Self‐Deployable and Foldable Origami Tent at Meter‐Scale

**FIGURE 5:**
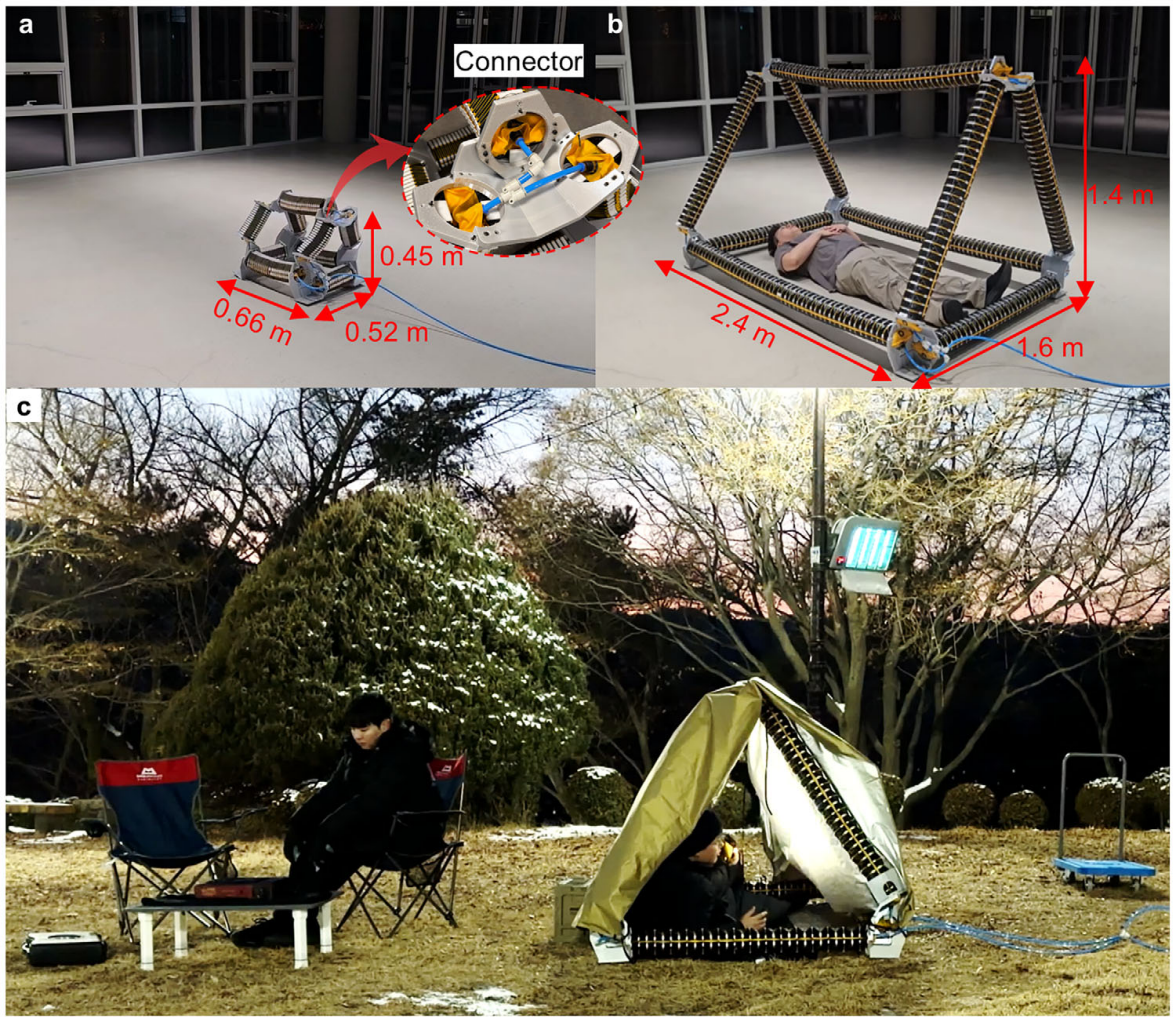
Self‐deployable and foldable origami tent. (a) Folded and (b) fully deployed state of the origami tent frame composed of three 2 m‐long and six 1.2 m‐long origami actuators. The tent exhibits 35‐fold 3D volumetric expansion upon deployment. (c) Origami tent used for outdoor camping.

To further highlight the scalability of our design method, we developed a self‐deployable and foldable origami tent frame (Figure 5). With the rapid growth of the global outdoor camping market, there is an increasing demand for automated tents that can be readily deployed with minimal human intervention. However, most of the existing products often employ pitching mechanisms or pneumatic deployment systems that enable automatic deployment only, while fully automated retraction remains largely unavailable. As a basis for realizing fully automated deployment and packing, we fabricated the origami tent frame consisting of three 2 m‐long and six 1.2 m‐long origami actuators with R = 50 mm, CT = 2.8 mm, and a deployment ratio of 3.96. For the 2 m‐long actuator, two sets of CSMs that correspond to 1 m‐long actuator were fabricated and assembled. All nine actuators were pneumatically linked to a common input line and can be simultaneously pressurized up to 100 kPa for deployment or depressurized to −20 kPa for retraction (Figure S10). 3D printed joints were also used to mechanically connect the origami actuators (Figure 5a, inset), and the total weight of the tent was measured to be 17.45 kg. In the retracted state, the tent occupies a compact volume of 0.52 × 0.66 × 0.45 m and deploys to 1.6 × 2.4 × 1.4 m, corresponding to a 35‐fold increase in 3D structural volume, providing sufficient space for two people (Figure 5b). Importantly, the tent can be deployed and retracted without any human intervention, as each origami actuator provides sufficient pulling force to overcome self‐weight and frictional resistance during the actuation (Figure 5c). When deployed outdoors, the deployment and retraction times were 420 and 360 s, enabling the users to focus on other tasks (Movie S7). When the system is deployed on smooth surfaces, full deployment and retraction times could be reduced to 60 and 170 s, respectively. Once deployed and the valves were closed, the tent can maintain its deployed configuration without additional energy input or active pressurization. This result highlights the potential of the proposed origami design framework for practical use in diverse engineering and societal applications.

## 3 Conclusions

The intrinsic limitation on the load‐carrying actuation in origami‐inspired mechanisms remains a challenge for practical robotic applications. We have shown that the assembly of the CSMs onto the origami actuator provides antagonistic shape retention force upon deformation, enabling stable robotic actuation while bearing external loads. Based on the Yoshimura origami kinematics and equilibrium analysis, our framework enables dynamic programming of robotic performances across a broad design space, including axial and transverse load capacity, deployment ratio, and scale. Importantly, the r2r FDM printing further enables successful production of meter‐scale origami actuators, which are otherwise challenging to achieve with existing approaches. To highlight this unique advantage, we developed a soft robotic manipulator capable of changing its workspace on demand while carrying the payload. The soft origami tent frame achieved autonomous deployment and contraction, indicating its potential for deployable shelter systems. However, the manual assembly of CSMs may present a challenge for large‐scale production. As the current assembly process involves attaching a thread to the end of each CSM and pulling it, this could be facilitated by using a motorized jig that pulls the thread. Although the printer used in this work (Bambu Lab H2S) can fabricate CSMs for origami actuators with a fully deployed length of up to 1.7 m, FDM printers with larger build platforms could further increase the CSM size to cover a longer actuator segment. In addition, there is also room for improvement in reducing the printing time and extending the printable width (i.e., the radius of the origami actuator). Scenario‐specific tests that directly reflect different loading conditions could also be beneficial for broader applications. Nevertheless, it is worth highlighting that the presented concept of modularized local creaseline stiffness control can be readily extended to different origami patterns such as Miura‐Ori (Figure S11). We envision that the proposed method offers a scalable and generalizable framework that facilitates the development of soft origami robots toward real‐world applications.

## 4 Experimental Section

### 4.1 R2R FDM 3D Printing of Pneumatic Origami Actuators

The r2r FDM 3D printer was developed based on a commercially available FDM 3D printer featuring a conveyor bed platform (CR30, Creality, China). The conveyor rollers included in the original system were replaced with r2r feeding of a flexible membrane to enable direct deposition of origami facets on the membrane via 3D printing. The standard FDM printers (Original Prusa i3 MK3S, Prusa Research, Czech Republic, and Bambu Lab H2S, Bambu Lab, China) were used to produce the CSMs. Both the origami facets and the CSMs were printed using a 0.8 mm‐ diameter nozzle. A commercially available TPU filament with a maximum tensile strain of 400% and a Shore hardness of 95A was obtained from eSUN Industrial Co., Ltd., Shenzhen, China. The tarpaulin sheet with an elastic modulus of 143 MPa and tensile strength of 25 MPa was selected as a membrane and obtained from Guangzhou Yuxin Plastic Co., LTD., China. The width and the thickness of the tarpaulin sheet were set to be 220 and 0.47 mm, respectively, according to the dimensions of the printing system. The printing parameters, including the printing temperature and speed, were adjusted to print the origami facet on the membrane. Specifically, the molten filament extruded from the nozzle is then deposited onto the membrane, partially melting its TPU‐coated surface. Therefore, a strong adhesion between the membrane and the printed facets can be achieved by slightly reducing the gap (∼0.1 mm) between the nozzle and membrane. The nozzle temperature and the bed temperature were heated to 240°C and 40°C, respectively, while the printing speed was set to 25 mm/s to ensure stable printing. The overhang printing speed was increased to 40 mm/s to produce the tunnel structure embedded in the facets. The positioning error of the r2r feeding mechanism was evaluated by measuring the offset between the two printed lines while advancing and re‐rolling the membrane. Ten trials were performed for each feeding distance (100 and 200 mm), yielding a mean positional error of < 0.8 mm for both conditions. The same parameters were used to 3D print the CSMs, while the printing speed was increased to 40 mm/s. The high‐frequency welding process for thermal bonding of the printed membranes was outsourced to a local fabrication workshop (Seoul, Korea), using an industrial welding machine (HS‐BT‐7500, HS Electronic) under a power of 5 kW and a welding time of 1.5 s. Pneumatic fittings (GPPM 10, Sanga Pneumatic, Seoul, Korea) were fastened onto the membrane of each origami actuator to interface them with an external pump and controller.

### 4.2 Pneumatic Control System

The custom pneumatic control system that combines a positive pressure regulator (ARP20‐02BG, SMC), negative pressure regulator (IRV20‐C06, SMC), solenoid valve (UV307‐4LL‐02, ShinYeong Mechatronics), positive pressure pump (EWS06, QuietZone), and vacuum pump () was developed. The origami actuator is connected to the exit port of the solenoid valve in the pneumatic system via pneumatic fitting (GPPM 10, Sang‐A Pneumatic). When the solenoid is turned to its normally on position, negative pressure is supplied to the origami; and when the solenoid is turned to its normally off position, positive pressure is supplied to the origami, pressurizing it up to 100 kPa for deployment. A pressure gauge (ISE30A‐01‐C‐M, SMC) is connected to the origami to measure the applied pressure simultaneously. The process was controlled using a custom LabVIEW script.

### 4.3 Experimental Setup for Origami Actuator Characterization

To test the axial payload of the origami actuator, a custom‐built testing setup was devised. As shown in Figure 3, the origami actuator was anchored at one end to a jig, whereas the other end was coupled to a moving block on a linear guide rail, allowing translational motion driven by pneumatic pressure and external force. A weight was connected and suspended to the moving block using a tendon and pully system. We used an OptiTrack visual tracking system (OptiTrack V120:Trio) to capture the origami folding trajectories and deformation, and reflective markers with a diameter of 14 mm were placed on each mountain fold for motion capture. The folding angle was then calculated by post‐processing the captured 3D coordinates with the developed kinematics model. To test the actuation capability under the transverse load, we utilized the same linear motion setup, while the weight was suspended at the center of the actuator (Figure 3d). As shown in Figure S5, the torsional stiffnesses of the CSMs were measured on a universal testing system (Model 34SC‐1, Instron).

### 4.4 Soft Origami Robotic Arm

The rotational motions in the yaw and pitch directions were driven by electric motors (H52‐100‐S500‐R, Robotis, South Korea), controlled a microcontroller (Arduino Mega 2560, Arduino) and a motor shield (RC‐100, Robotis). A commercially available suction cup with a diameter of 30 mm (VFBL30, F.TEC) was mounted at the distal end of the robotic arm. To mechanically integrate the origami actuators, custom connectors were fabricated using conventional FDM printing (Original Prusa i3 MK3S, Prusa Research, Czech Republic). The collision safety of the robotic arm was evaluated based on ISO/TS 15066. The end effector was allowed to collide with a pressure measurement pad (model), while its velocity was recorded using an OptiTrack visual tracking system (OptiTrack V120:Trio).

## Author Contributions

**Sung Yol Yu**: conceptualization, methodology, data curation, investigation, validation, formal analysis, visualization, writing – original draft, writing – review and editing. **Sang‐Joon Ahn**: conceptualization, methodology, investigation, validation, formal analysis, data curation, writing – review and editing, writing – original draft, visualization. **Wonchul Lee**: visualization, methodology, investigation. **Hyun‐Jin Hong**: visualization, investigation. **Seunguk Moon**: methodology, investigation. **Kyu‐Jin Cho**: conceptualization, funding acquisition, writing – review and editing, supervision, resources, project administration.

## Conflicts of Interest

The authors declare no conflicts of interest.

The data that support the findings of this study are available from the corresponding author upon reasonable request.

## Supporting information




**Supporting File 1**: advs77127‐sup‐0001‐SuppMat.docx.

**Supporting File 2**: advs77127‐sup‐0002‐MovieS1.mp4.

**Supporting File 3**: advs77127‐sup‐0003‐MovieS2.mp4.

**Supporting File 4**: advs77127‐sup‐0004‐MovieS3.mp4.

**Supporting File 5**: advs77127‐sup‐0005‐MovieS4.mp4.

**Supporting File 6**: advs77127‐sup‐0006‐MovieS5.mp4.

**Supporting File 7**: advs77127‐sup‐0007‐MovieS6.mp4.

**Supporting File 8**: advs77127‐sup‐0008‐MovieS7.mp4.
